# **Cyclodextrine Screening for the Chiral Separation of Carvedilol by Capillary Electrophoresis**

**Published:** 2015

**Authors:** Gabriel Hancu, Anca Cârje, Ileana Iuga, Ibolya Fülöp, Zoltán-István Szabó

**Affiliations:** a *Department of Pharmaceutical Chemistry, Faculty of Pharmacy, University of Medicine and Pharmacy, Târgu Mureş, Romania.*; b *Department of Analytical Chemistry, Faculty of Pharmacy, University of Medicine and Pharmacy, Târgu Mureş, Romania.*; c *Department of Toxicology and Biofarmacy, Faculty of Pharmacy, University of Medicine and Pharmacy, Târgu Mureş, Romania.*

**Keywords:** Carvedilol, Capillary electrophoresis, Chirality, Cyclodextrines, Enantiomer separation

## Abstract

Carvedilol is administered as a racemic mixture of the R(+)- and S(-)-enantiomers, although it was demonstrated that the two enantiomers exhibit different pharmacological effects and stereoselective pharmacokinetics. The aim of this study was the evaluation of several native and derivatized cyclodextrines as chiral selectors for the separation of carvedilol enantiomers. Stereoselective interactions were observed with four cyclodextrines (*β*-CD, hydroxypropyl-*β*-CD, randomly methylated *β*-CD and sulfobuthyl ether- *β*-CD). The effects of CD concentration, pH value and composition of the background electrolyte, capillary temperature, running voltage and injection parameters have been investigated. The method was validated for precision of peak-area response, linearity range and limits of detection and quantification. An efficient stereoselective capillary zone electrophoretic method was developed for the determination of carvedilol enantiomers using a simple 25 mM phosphate buffer at a pH = 2.5 and 10 mM *β*-CD as chiral selector, resulting in baseline separation of the two enantiomers with sharp peaks and relatively short analysis time. Highly satisfactory results were obtained from the analysis of carvedilol from tablets, indicating that the method is suitable for routine analysis of carvedilol in pharmaceutical products.

## Introduction

Carvedilol is a non-cardioselective *β*-blocker, which also exhibit *α*-blocking activity. It has vasodilating properties, attributed mainly to its blocking activity on *α* receptors; but at higher doses calcium-channel blocking activity may contribute also. Carvedilol is reported to have no intrinsic sympathomimetic activity and only have weak membrane-stabilizing activity (1).

Carvedilol is used not only in the management of hypertension and angina pectoris, but also in the therapy of symptomatic heart failure. This later indication is unique amongst available *β*-blockers, for which this condition is normally a contraindication. Carvedilol utility in congestive heart failure can be explained due to the combination of decreased vascular resistance (*α*-adrenergic antagonism) and lack of reflex tachycardia ( -adrenergic antagonism) (1).

**Figure 1:**
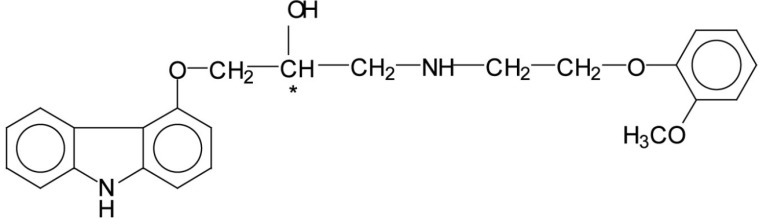
The chemical structure of carvedilol. The asterix denote the chiral center.

Like all other *β*-blockers that are currently used in clinical practice, carvedilol contains an asymmetric carbon atom in the amino-alkanol side chain resulting in the existence of two enantiomers. The chemical structure of carvedilol (1-(4-carbazolyloxy)-3-(2-(2-methoxy) ethyl-amino)-2-propanol) is presented in Figure 1.

The d-enantiomer of carvedilol shows the (R)-configuration while the l-enantiomer shows the (S)-configuration (2).

Similar to other *β*-blockers, the S(–) enantiomer of carvedilol is more potent (200-fold higher) as an antagonist of *β*-receptors than the R(+) enantiomer. When *β*-blocking effects of the R(+) and S(-) enantiomers have been compared, the results were similar both *in-vitro* and in human studies, revealing that the S(-) enantiomers are markedly more effective. Both the R(+) and S(-) enantiomers, however, are equally effective in blocking *α-*adrenergic activity (2,3).

Although its two enantiomers have both different pharmacodynamics and pharmacokinetic properties, carvedilol is marketed as a racemic mixture. Furthermore, plasma concentrations of the two enantiomers differ significantly and in wide ranges when the racemic mixture is administered, as plasma concentrations of the R(+) enantiomer are higher than those of the S(–) enantiomer. Also the degree of stereoselectivity in the pharmacokinetics of carvedilol is susceptible to change according to the patient and/or disease characteristics (2).

Metabolism of carvedilol is also stereoselective giving rise to different bioavailabilities of the enantiomers of about 15% for S(–)-carvedilol and about 31% for R(+)-carvedilol; while, terminal elimination half-life for R(+)-carvedilol ranges from 5 to 9 hours, compared with 7 to 11 hours for S(–)-carvedilol (4,5).

Taking in consideration all these aspects mentioned above elaboration of new methods for the chiral separation of carvedilol enantiomers becomes a necessity but also a challenge.

High performance liquid chromatography (HPLC) methods using chiral derivatization with an optically pure derivatization reagent followed by separation of the two diasteromers using an achiral method, have been developed, however these methods can be rather expensive and sometimes an incomplete reaction and multiple derivatization could occur (6).

Meanwhile capillary electrophoresis (CE) has obtained increasing importance in analytics, especially in the separation of chiral compounds, including here the enantioseparation of carvedilol (5). Several reports comparing HPLC and CE methods used for the chiral separation of carvedilol enatiomers have shown that CE is a more effective regarding chiral resolution and analysis time, except detectability (4,7).

The advantages of CE compared to conventional HPLC methods in the enantioseparation of chiral substances are being related to the: rapid and simple method development, small amounts of solvent, sample and chiral selector required for the separation and especially with the high selectivity in choosing and changing the chiral selector (8).

However, the optimal separation conditions for the CE chiral separation of carvedilol enantiomers are significantly different in several cases, and the basis of the differences has not been clearly identified (9,10).

The most widely used chiral selectors in CE are the cyclodextrins (CDs), cyclic oligosaccharides having an external hydrophilic surface and a hydrophobic cavity, in which they can include other compounds by hydrophobic interaction. The inclusion mechanism is sterically selective because analytes must ﬁt the size of the cavity, the diameter of which depends on the number of glucose units in the CD structure. Interaction with an analyte will occur by inclusion of its hydrophobic portion in the cavity and from hydrogen bonding to the chiral hydroxyl groups of the rim. In CE chiral separation can be achieved by adding CDs directly to the buffer electrolyte (11).

The large majority of the published studies in this field, are dealing with the separation of beta-blockers in general or with the separation of carvedilol using only a particular CD derivative, and not with the screening of several CD derivative and the optimization and understanding of the separation process, in order to evaluate the optimum chiral selector for the enantioseparation (4,5,9,10).

Our aim was to develop an alternative rapid, simple and efficient method for the chiral separation of carvedilol enantiomers using a systematic screening approach of different native and derivatized CDs as chiral selectors and the optimization of electrophoretic conditions in order to obtain a good chiral resolution in a short analysis time.

## Experimental

*Chemicals*

R,S–carvedilol, R-carvedilol and S-carvedilol of pharmaceutical grade were purchased from Moehs Productos Quimicos (Barcelona, Spain). The internal standard S-propranolol was also acquired from the same producer. For the determination of carvedilol from commercial pharmaceuticals we used Carvedilol (Sandoz, Romania) tablets containing 25 mg carvedilol. The following reagents of analytical grade were used: phosphoric acid (Pernix Pharma, Hungary), methanol, sodium hydroxide (Lach Ner, Czech Republic), sodium tetraborate, disodium hydrogenphosphate, sodium didydrogenphosphate (Merck, Germany). Purified water was provided by a Milli-Q Plus water purification system (Millipore, USA).

As chiral selectors we used the following CD derivatives of research grade: native neutral CDs (*α*-CD, *β*-CD, γ-CD), derivatized neutral CDs (hydroxypropyl-*β*-CD - HP-*β*-CD, randomly methylated *β*-CD – RAMEB), anionic substituted charged CD (sulfobuthyl ether- *β*-CD sodium salt – SBE-*β*-CD). All CDs were obtained from Cyclolab (Budapest, Hungary) with the exception of SBE-*β*-CD - Capsitol (Cydex, USA).

*Instrumentation*

μuncoated fused silica-capillaries (Agilent, Germany). The electropherograms were recorded and processed by Chemstation 7.01 (Agilent, Germany) software. The pH of the buffer solutions was determined with the Terminal 740 pH–meter (Inolab, Germany).

*Sample preparation*

μ^-1^ and later diluted with the same solvent to the appropriate concentration. The samples were introduced in the system at the anodic end of the capillary by hydrodynamic injection. Buffer solutions were prepared in water; the pH was adjusted with sodium hydroxide. All samples and buffers were ﬁltered with a 0.45 μm ﬁlter and degassed in an ultrasonic bath for 5 minutes before use.

For the determination of carvedilol from tablets, twenty Carvedilol tablets were weighed, and the medium net weight of each tablet was calculated. The tablets were powdered in a mortar and an amount of powder equivalent to the average weight of a tablet was weighed and dissolved in methanol, and the solution was diluted to 100 mL in a volumetric flask and sonicated for 10 minutes. The samples of the tablet solution were centrifuged at 3500 rpm for 10 minutes. The supernatant was diluted, following the same procedure as for the preparation of standard solutions, before CE separation.

*Separation conditions and methodology*

The capillaries were conditioned before use with 0.1 M sodium hydroxide for 30 minutes and with the background electrolyte used in the analysis for 30 minutes. The capillary was rinsed for 1 minute with 0.1M sodium hydroxide and buffer solutions before each electrophoretic separation.

We recorded previously the UV spectra of carvedilol and found absorption maximum in methanol at 242 nm, which was elected as detection wavelength in the CE separations. We applied some “standard” electrophoretic conditions for a CE analysis: temperature 20 ˚C, applied voltage + 20 kV, injection pressure/time 50 mbar/3 sec., sample concetration 10 μg mL^-1^.

In the initial experiments the studied compounds were injected in the absence of CDs and their effective mobility was calculated. Then we performed the measurements using the same BGE, containing a relatively low amount of chiral selector in order to verify the decrease in the effective mobility of the analytes. We applied a direct chiral separation method by simply dissolving the optically pure additive in the selected buffer solution.

α_2_ - t_1_)/(w_1_ + w_2_) equation, where the migration times (t_1 _and t_2_) and the peak-widths at the baseline (w_1_ and w_2_) were marked for the slow and fast migrating enantiomers, respectively.

We also evaluated the migration retardation factor (R_m_) defined as the ratio of the migration time of the analyte (if resolved, of the second eluted enantiomer) in the buffer containing the chiral selector and the migration time of the analyte in the plain buffer; which represents an approximate parameter for measuring the strength of the host–guest interaction.

## Results and Discussions

*Preliminary study*

In order to find the suitable conditions for the chiral separation of carvedilol, a series of preliminary experiments were conducted at different pH and buffer compositions. In the preliminary analysis we used 25 mM phosphoric acid (pH = 2.1), 25 mM disodium hydrogenphosphate – 25 mM sodium didydrogenphosphate (pH = 7) and 25 mM sodium tetraborate (pH = 9.3) background electrolytes (BGEs) respectively and modified the buffer pH by adding a 0.1M sodium hydroxide solution.

In comparison with other commonly used *β*-blockers (pK_a_ values around 9-9.5) carvedilol has a pK_a_ value of 7.97; the explanation of this discrepancy being attributed especially to the inductive effect of the *β*-*O*-atom, which lowers the basicity of the amino group (12).

For a basic drug like carvedilol with a pK_a_ > 7.0 its net charge at a pH between 2 and 5 is not significantly different, showing that analyte charge is insensitive to pH. It is, nevertheless, well known that the electroosmotic flow (EOF) is sensitive to pH in the range between 3.0 and 7.0; as it decreases considerably with decreasing pH. The migration time of carvedilol increased when the pH of the buffer increased from 2 to 5, and decreased as the pH was increased from 5 to 7.

The most suitable BGE proved to be a 25 mM phosphoric acid solution at a pH of 2.5; using this buffer we obtained well-shaped peak for carvedilol with a migration time of 6.8 minutes. At this low pH the effect of the EOF is minimal, and the analyte will migrate mostly through its own electrophoretic mobility.

Method optimization

Buffer ionic strength, pH, CD type and concentration, as well as applied voltage, temperature and injection parameters were optimized.

The first requirement for inclusion complexation is fitting the analyte into the CD cavity, thus selecting the appropriate CD is related to the shape and dimension of the analyte.

Several CDs (native and derivatized) were tested in order to obtain chiral resolution of carvedilol enantiomers. Initial concentration of 10 mM neutral CDs were added to the buffer solution, while for charged CDs we added a concentration of 5 mM in order to limit the increase of ionic strength which generated high currents and instability of the electrophoretic system.

No chiral separation only an increase of the migration time was observed when using *α*-CD and γ-CD. Clearly *α*-CD was not able to separate the carvedilol racemic mixture because its cavity is too small, *β*-CD allowed chiral resolution while γ-CD was not able to guest the studied analyte probaby because carvedilol molecule is too small. Stereoselective interactions of the two enatiomers were observed also in the case of *β*-CD derivatives, HP-*β*-CD and RAMEB; as substitution at the secondary hydroxyl rim on the surface of the CD can affect selectivity of the separation, as it will provide additional interaction points with the analyte.

**Figure 2:**
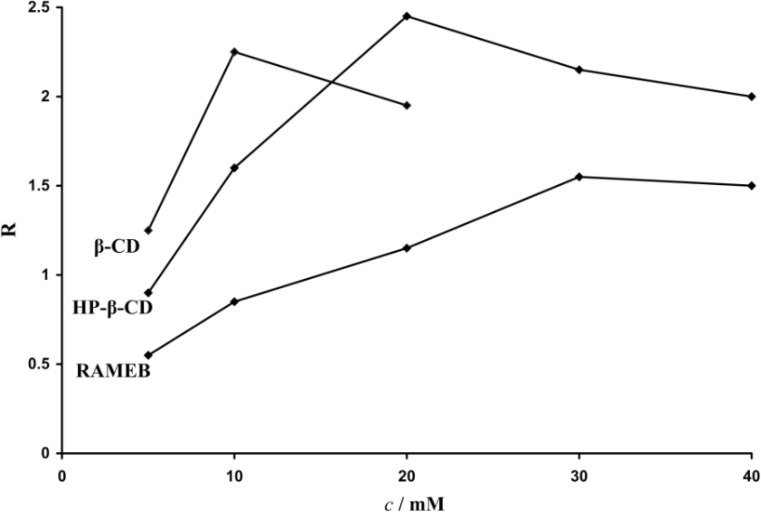
Effect of CD concentration on the chiral resolution (electrophoretic conditions: BGE 25 mM phosphoric acid, pH = 2.5, voltage + 20 kV, temperature 20 ˚C, hydrodinamic injection 50 mbar/3 s., UV detection 242 nm).

If complexation was observed but insuficient resolution was achieved, we increased the concentration of the chiral selector (5-40 mM) until satisfactory separation was obtained. When using *β*-CD as chiral selector the maximum concentration was 20 mM, due to its poor solubility in aqueous solutions. Resolution of the enantiomers was calculated and the optimal concentration for the separation was determined. Figure 2 shows the effect of chiral selector concentrations on the carvedilol enantiomers separation.

The CD concentration plays a very important role in the chiral resolution, and should be carefully controlled in order to find the optimal experimental conditions. The optimal concentration depends on the binding affinity of the two enantiomers with the chiral selector. The migration times increased with an increase in CDs concentration. This is due to longer residence time of the analyte in the complex form as well as to an increase in the viscosity of the buffer with a reduction of the mobility of the analytes.

An increase in the pH value of the BGE increased migration times but no direct correlation due to increase or decrease of the pH on the chiral resolution was obtained. At low pH values there is more time for the analyte to interact with the CD to result in increased time spent in the capillary; while at pH values above 5 where the effect of the EOF becomes more significant, the migration times of the analytes increased. Low operational pH was found to be essential for the resolution of carvedilol enantiomers. A buffer pH of 2.5 was elected for the separation.

The use of an ionized CD (SBE-*β*-CD) resulted in long migration times (above 20 minutes) but only a small peak splitting and also a severe peak tailing was observed at a pH of 2.5. At this pH, SBE-*β*-CD is negatively charged while carvedilol is positively charged, consequently SBE-*β*-CD moves towards the anode while carvedilol towards the cathode (13,14).

The use of a dual CD system, which include a combination of a neutral and a charged CD (10 mM *β*-CD + 5 mM SBE-*β*-CD) led to an increase of migration times but only a small peak splitting has been observed.

But, using a phosphate buffer solution with a pH of 7 and 5 mM SBE-*β*-CD as chiral selector led to the baseline chiral separation of the two enantiomers and a very fast separation time (5 minutes), the two enantiomers migrating faster than the EOF. An increase in the SBE-*β*-CD concentration didn’t increase resolution of the separation.

The chemical composition and the concentration of the buffer can affect the baseline stability, peak shape and separation selectivity. The enantiomer separation using phosphoric acid as BGE was better than those compared with disodium hydrogenphosphate – sodium didydrogenphosphate and sodium tetraborate buffers. A slight increase in the migration time of the analyte was observed with increasing buffer concentration, but only little inﬂuence on the separation could be observed. Hence a 25 mM phosphoric acid buffer was elected for the enantioseparation.

Addition of an organic modiﬁer such as methanol or acetonitrile to the phosphate buffer resulted in longer migration times but there was no significant improvement in the chiral separation.

Running voltage did not have a strong effect on the resolution; while a decrease in temperature led to extension in analysis time and to a slight increase of the chiral resolution. An optimal voltage of + 20 kV and a temperature of 15 ^0^C was elected, in order to obtain an adequate resolution of the separation and a satisfactory analysis time.

A high injection pressure of 50 mbar and a fast injection time of 1 second provided a reasonable sample load and maintained resolution. The amount injected was enough to achieve a sufficient signal/noise ratio but not large enough to cause band broadening.

The electrophoretic mobility of carvedilol stereoisomers decreased with increasing capillary length because of the longer migration time to the detection window.

The migration order of the two enantiomers was determined by injecting a solution of the racemate enriched with the pure enantiomer separately. The ﬁrst peak to pass the detector window was determined to be R(+) carvedilol followed by S(-) carvedilol.

Table 1 summarizes the experimental conditions (concentration and type of chiral selector, pH, voltage, temperature) and the results (migration times of the separated enantiomers, separation factor, resolution) obtained for the CDs that exhibited chiral interactions with carvedilol enantiomers.

**Table 1:** Capillary electrophoretic separation of optical isomers of carvedilol using cyclodextrin derivatives as chiral selectors (BGE 25 mM phosphoric acid, pH – 2.5)

CD-derivative	**pH**	**Voltage / kV**	**Temperature / ** ^0^ **C**	**t**_1_ ** / min**	**t**_2 _ **/ min**	**R**_m_		**R**
10 mM *β*-CD	2.5	20	15	11	11.5	1.69	1.04	2.74
20 mM HP-*β*-CD	2.5	20	15	12.9	13.5	1.98	1.04	2.54
30 mM RAMEB	2.5	20	15	15.4	15.9	2.32	1.03	1.98
5 mM SBE-*β*-CD	2.5	20	15	21.2	21.6	3.17	1.03	0.83

Taking in consideration the aspects presented above we can conclude that the optimal electrophoretic conditions for the carvedilol enantioseparation are: BGE: 25 mM phosphoric acid chiral selector: 10 mM *β*-CD, buffer pH = 2.5, applied voltage: + 20 kV, temperature: 15 ^0^C, injection pressure/time: 50 mbar/1 s, UV detection at 242 nm. Also HP-*β*-CD (20 mM) or RAMEB (30 mM) can be used successfully as chiral selectors in the enantioseparation of carvedilol, with good chiral resolutions but longer migration times.

**Figure 3:**
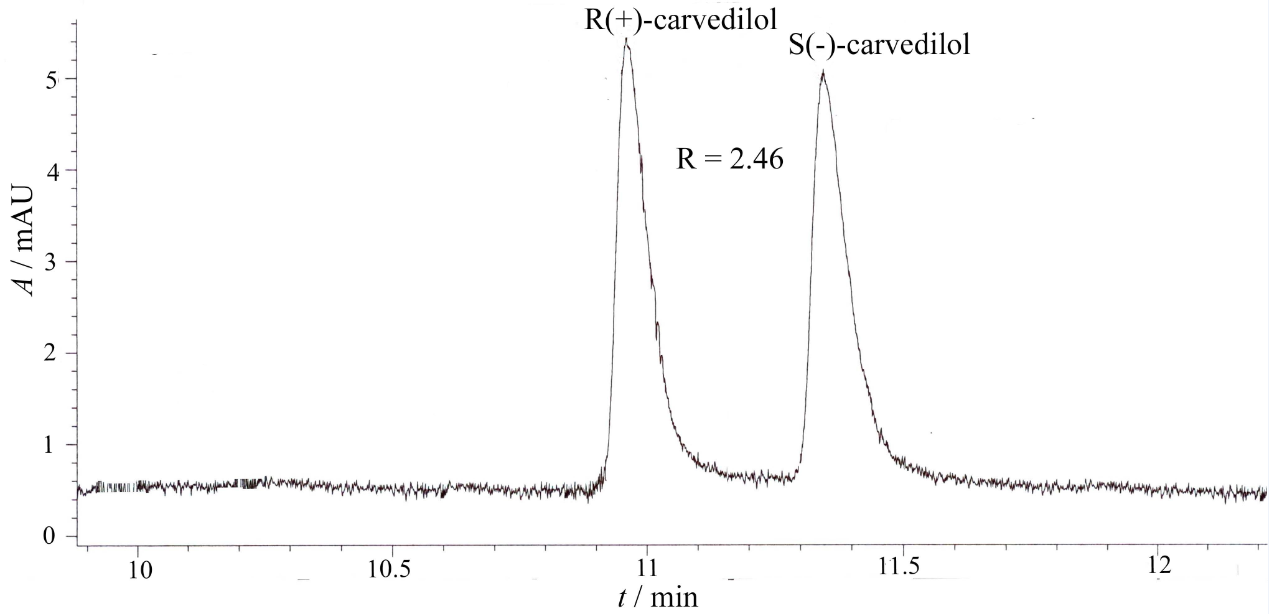
Capillary electrophoretic separation of carvedilol enantiomers using *β*-CD as chiral selector (experimental conditions: BGE 25 mM phosphoric acid, chiral selector 10 mM *β*-CD, pH = 2.5, voltage + 20 kV, temperature 15 ^0^C, hydrodinamic injection 50 mbar/1 sec., sample concentration 10 μg/mL, UV detection 242 nm

**Figure 4:**
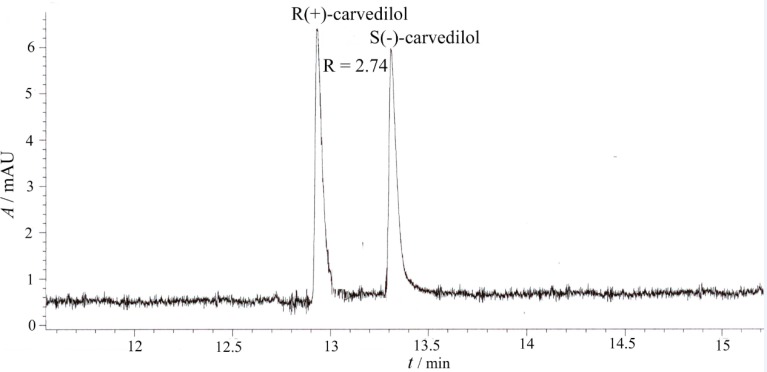
Capillary electrophoretic separation of carvedilol enantiomers using HP-*β*-CD as chiral selector (experimental conditions: BGE 25 mM phosphoric acid, chiral selector 20 mM HP-*β*-CD, pH = 2.5, voltage + 20 kV, temperature 15 ^0^C, hydrodinamic injection 50 mbar/1 sec., sample concentration 10 μg/mL, UV detection 242 nm)

The electropherograms of the enantiosepartion using *β*-CD and HP-β-CD as chiral selectors are presented in Figure 3 and Figure 4.

*Analytical parameters*

The pure enantiomer of another *β*-blocker, S-propranolol was used as an internal standard (IS); its migration time being faster than the one of R(+)-carvedilol. Quantiﬁcation was accomplished on the basis of carvedilol enantiomer to IS peak-area ratios (peak area of carvedilol enantiomer/peak area of IS).

Calibration plots were constructed by preparing standard solutions (n = 3) at six concentrations in a specific concentration range (concentration range: 2.5 - 50 µgmL^-1^), and showed good linearity (Table 2). High correlation coefficients were obtained and the intercepts of the plots were not signiﬁcantly different from zero.

The limits of detection (LOD) and quantiﬁcation (LOQ) were estimated as: standard deviation of regression equation/slope of the regression equation multiplied by 3.3 and 10, respectively (Table 2).

**Table 2:** Calibration data and LOD/LOQ values for carvedilol chiral separation (calibration range: 2.5 - 50 µg mL^-1^).

**Enantiomer**	**Regression equation**	**Correlation coefficient**	**LOD/µgmL** ^-1^	**LOQ/µgmL** ^-1^
**R(+) carvedilol**	y = 0.4625x + 0.8491	0.992	**1.13**	**3.43**
**S(-) carvedilol**	y = 0.4465x + 0.8177	0.997	**1.18**	**3.57**

The precision of the method was determined by measurement of repeatability (intra-day) and intermediate precision (inter-day), expressed as RSD (relative standard deviation) % for a series of measurements.

Standard solutions of carvedilol and internal standard were prepared in methanol and injected on three consecutive days, six times a day (Table 3).

**Table 3:** Intra and inter-day repeatability of carvedilol to IS peak-area ratio (n-number of experiments; k-day of experiment; RSD / % -relative standard deviation; sample, IS concentration - 10 µgmL^-1^).

**Enantiomer**	**Day 1 n=6**	**Repeatability** **day 2 n=6** **RSD / %**	**Day 3 n=6**	**Intermediate** **precission n=6, k=3**
**R(+) carvedilol**	1.14	1.68	**1.96**	**1.98**
**S(-) carvedilol**	1.18	1.88	**2.04**	**2.02**

A concentration of 10 µgmL^-1^ was used to evaluate precision as degree of repeatability by performing six replicate analyses and calculating the coefficient of variation, the RSD values were 1.35% for R(+) carvedilol and 1.36% for S(-) carvedilol, respectively; these results showing that the method is sufficiently precise.

±±

## Conclusions

In general, the enantiomers of the *β*-adrenergic antagonists share similarities with respect to pharmacologic effects, with the S(–)-enantiomer usually possessing a substantially higher ability to bind to *β*-adrenergic receptors. In the case of carvedilol the enantiomers may posses also other useful pharmacological qualities that add to the *β*-blocking properties of the drug. Therefore, maybe the time has arrived that the optically pure enantiomers should be recognized as distinct drugs; hence the racemates can no longer be regarded as optimal for patients on *β*-blocker therapy, as R(+)-enantiomers that make up 50% of every racemic *β*-blocker may contribute to an increase of side effects as well as to drug interactions.

CD type and concentration, pH value of the BGE had a strong influence on the efficiency of the chiral separation. The changes in the concentration of the CDs and in the pH of the BGE showed uneven effect on the resolution of the optical isomers.

The faster migration of the R(+) carvedilol indicates that this enantiomer have weaker interaction with the CDs, while the interaction of S(-) carvedilol has a stronger one. Previous studies involving other *β*-blockers reported the faster migration of the S enantiomers, but in the case of carvedilol the changing in the migration order of the enantiomers only reflects the particularities in its chemical structure (10).

Among the analyzed CDs, *β*-CD, HP-*β*-CD and RAMEB showed baseline separation of the two enantiomers, while the use of SBE-*β*-CD led only to partial separation. The best results were obtained using a 25 mM phosphate buffer at a pH = 2.5 and 10 mM *β*-CD as chiral selector.

In conclusion, an efficient stereoselective CE method was developed for the determination of carvedilol enantiomers using a simple phosphate buffer and CDs as chiral selectors, resulting in baseline separation of the two enantiomers with sharp peaks and relatively short analysis time.
